# Spatial genetic structure, genetic diversity and pollen dispersal in a harvested population of *Astrocaryum aculeatum* in the Brazilian Amazon

**DOI:** 10.1186/s12863-016-0371-8

**Published:** 2016-04-23

**Authors:** Santiago Linorio Ferreyra Ramos, Gabriel Dequigiovanni, Alexandre Magno Sebbenn, Maria Teresa Gomes Lopes, Paulo Yoshio Kageyama, Jeferson Luis Vasconcelos de Macêdo, Matias Kirst, Elizabeth Ann Veasey

**Affiliations:** Escola Superior de Agricultura “Luiz de Queiroz”/Universidade de São Paulo (ESALQ/USP), Departamento de Genética, C.P. 9, CEP 13418-900 Av. Pádua Dias, 11, Piracicaba, SP Brazil; Instituto Florestal de São Paulo, Seção de Melhoramento e Conservação Genética Florestal, C.P. 1322, CEP 01059-970 São Paulo, SP Brazil; Faculdade de Ciências Agrárias, Universidade Federal do Amazonas (UFAM), CEP 60077-000 Manaus, AM Brazil; ESALQ/USP, Departamento de Ciências Florestais, Av. Pádua Dias, 11, C.P. 9, CEP 13418-900 Piracicaba, SP Brazil; Embrapa Amazônia Ocidental, C.P. 319, CEP 69048-660 Manaus, AM Brazil; University of Florida, School of Forest Resources & Conservation, PO Box 110410, Gainesville, FL 32611-0410 USA

**Keywords:** Coancestry coefficient, Effective size, Gene flow, Microsatellites, Paternity analysis, Population genetics, Genetic structure, Tucumã of amazonas

## Abstract

**Background:**

*Astrocaryum aculeatum* is a palm tree species native to the tropical regions of South America, exploited commercially by local farmers for the pulp extracted from its fruits. The objective of this research was to compare the genetic diversity between adult plants and seedlings from open-pollinated seeds, quantify the pollen flow and dispersal, the spatial genetic structure, and the effective size of a population that has been continuously harvested for its fruits. The study was carried out in a natural population of *A. aculeatum* distributed over approximately 8 ha in the State of Amazonas (Brazil), separated by 400 m from the closest neighboring population. In total, 112 potential pollen donors, 12 mother plants and 120 offspring were mapped and genotyped.

**Results:**

Genetic diversity was high for parents and the offspring. The fixation indexes for adults (*F* = -0.035) and offspring (*F* = -0.060) were negative and not significant. A significant spatial genetic structure was detected for the adult plants (up to the distance of 45 m) indicating short-distance seed dispersal. Paternity analysis detected 9.2 % of pollen immigration and the average distance of pollination within the population was 81 m. The average effective pollination neighborhood area between plants was 1.51 ha.

**Conclusions:**

Our results indicate that substantial introduction of new alleles has occurred in the population through pollen immigration, contributing to the maintenance of genetic diversity. Conservation efforts aimed at maintaining the gene pool of the current population or establishing new populations should utilize offspring from mother plants selected to be spaced by at least 50 m to prevent collecting seeds from relatives.

## Background

Palm trees are considered one of the most useful groups of plants, especially for rural communities to which they provide building material, fabric, fuel, food, ornamental and medicinal plants [1]. The palm tree *Astrocaryum aculeatum* (Arecaceae) is found in the tropical forest of the Brazilian Amazon region. It is a monoecious species, morphologically showing an inflorescence that has the two floral structures (male and female) and that presents protogyny. Popularly known as tucumã of Amazonas, it is of significant economical importance for the populations of the Amazon region [2]. The pulp from the fresh fruit is directly consumed and also used to extract oil for the cosmetic industry and human consumption. The seed endocarp is used to make crafts [3, 4]. The species is adapted to non-flooded areas and is more frequently found in deforested areas or in areas that underwent anthropic action [3]. The distribution of *A. aculeatum* is restricted to the Western and Central Brazilian Amazon region, more specifically in the states of Acre, Mato Grosso, Rondônia, Roraima, part of Pará [4] and Amazonas. Amazonas is probably its most important center of genetic diversity [5]. Commercial harvest of *A. aculeatum* fruit is almost entirely dependent on extractivism. Consequently, highly irregular amounts of fruits of variable quality are commonly harvested. The growing market demand and the high price of the fruit has motivated farmers in the state of Amazonas to increasingly exploit commercially these plants, but the genetic consequences on natural populations has been unknown. Highly informative co-dominant microsatellite molecular markers [6] can be applied to measure the impact of current harvesting methods on natural populations of *A. aculeatum*. In particular, these markers can be used to measure the degree of genetic variation within and between populations, supplying important answers in population genetics and in ecological and evolutionary approaches [7], as well as estimating genetic diversity, endogamy, spatial population structure, mating system, and gene flow [8].

Gene flow in trees comprises both pollen and seeds dispersal [9, 10]. The male gametes are pollen-dispersed and the embryos carrying contributions from both parents are seed-dispersed [11]. Pollen and seed dispersal patterns significantly influence the genetic structure and the effective population size [12]. High levels of gene flow allow the maintenance of the genetic cohesion between populations whereas low levels result in genetic differentiation through genetic drift and local selection [13]. Studies based on genetic markers have shown that pollen can be dispersed over long distances in animal pollinated tree species [14, 15], preventing possible effects of genetic drift, such as loss of alleles, reduction in heterozygosity, increase in inbreeding and decrease in effective population size within populations [16].

Here we present a population genetics study of a harvested natural population of *A. aculeatum* established in the Brazilian Amazon region. The objectives of this study were to quantify the genetic diversity in adult individuals and open-pollinated seeds, quantify the pollen flow and dispersal, the spatial genetic structure, and the effective population size of this population. Estimating the effective number of pollen donors and the effective size of a natural population defines the minimum distance required between seed trees to avoid genetic relatedness in seed collections. This information is critical for conservation and tree breeding programs [14]. The associated parentage analysis methods [17] determined through categorical likelihood paternity analysis [18] is also important for allowing the genealogical reconstruction of the relatedness between individuals within and among progeny [14]. In summary, estimating parameters of genetic diversity, reproduction system, gene flow, pollen dispersal, and spatial genetic structure in harvested populations of *A. aculeatum* are fundamental for the establishment of adequate strategies for the use of this genetic resource.

## Results

### Genetic diversity

A set of 12 microsatellite loci was used in this study, of which two (Aac01 and Aac13) were monomorphic in the population and excluded from further analysis. A total of 81 alleles were identified in the ten polymorphic loci, distributed among all 244 sampled plants (adults + offspring). In adults, the total number of alleles per locus varied from 3 to 19, with a mean of 7.7. In offspring, the total number of allele was lower, varying from 2 to 14, with a mean of 6.4 (Table 1). Adults had also more private alleles (17) than offspring (4), suggesting low pollen immigration from other areas or recent population establishment. The observed and expected heterozygosity of the adults (*H*_*o*_ = 0.566; *H*_*e*_ = 0.547) were marginally lower than the offspring (*H*_*o*_ = 0.594; *H*_*e*_ = 0.560). The mean fixation index (*F*) was negative and not significantly different from zero for adults (-0.035) and offspring (-0.060), indicating absence of inbreeding.

**Table 1:** Genetic diversity indexes^a^ for adults and offspring in an *Astrocaryum aculeatum* population

Sample	*n*	*k*	*A*_*p*_	*A*	*H*_*e*_	*H*_*o*_	*F*	*P*_*2p*_
Adults	122.7	77	17	7.7 ns	0.547 ns	0.566 ns	−0.035 ns	0.996080
Offspring	118	64	4	6.4 ns	0.560 ns	0.594 ns	−0.060 ns	-

^a^ *n* sample size, *k* total number of alleles over loci, *A*_*p*_ number of private alleles in each generation, *A* is the average number of alleles per loci, *H*_*e*_ and *H*_*o*_ expected and observed heterozygosities, respectively, *F* fixation index, *P*_*2p*_ theoretical combined non exclusion probability for second parent. ^b^ *ns* non significant by the Student’s t- test with p (probability). *A*, *p* = 0.543; *H*_*e*_, *p* = 0.885; *H*_*o*_, *p* = 0.809; *F*, *p* = 0.894

### Spatial genetic structure

**Fig. 1:**
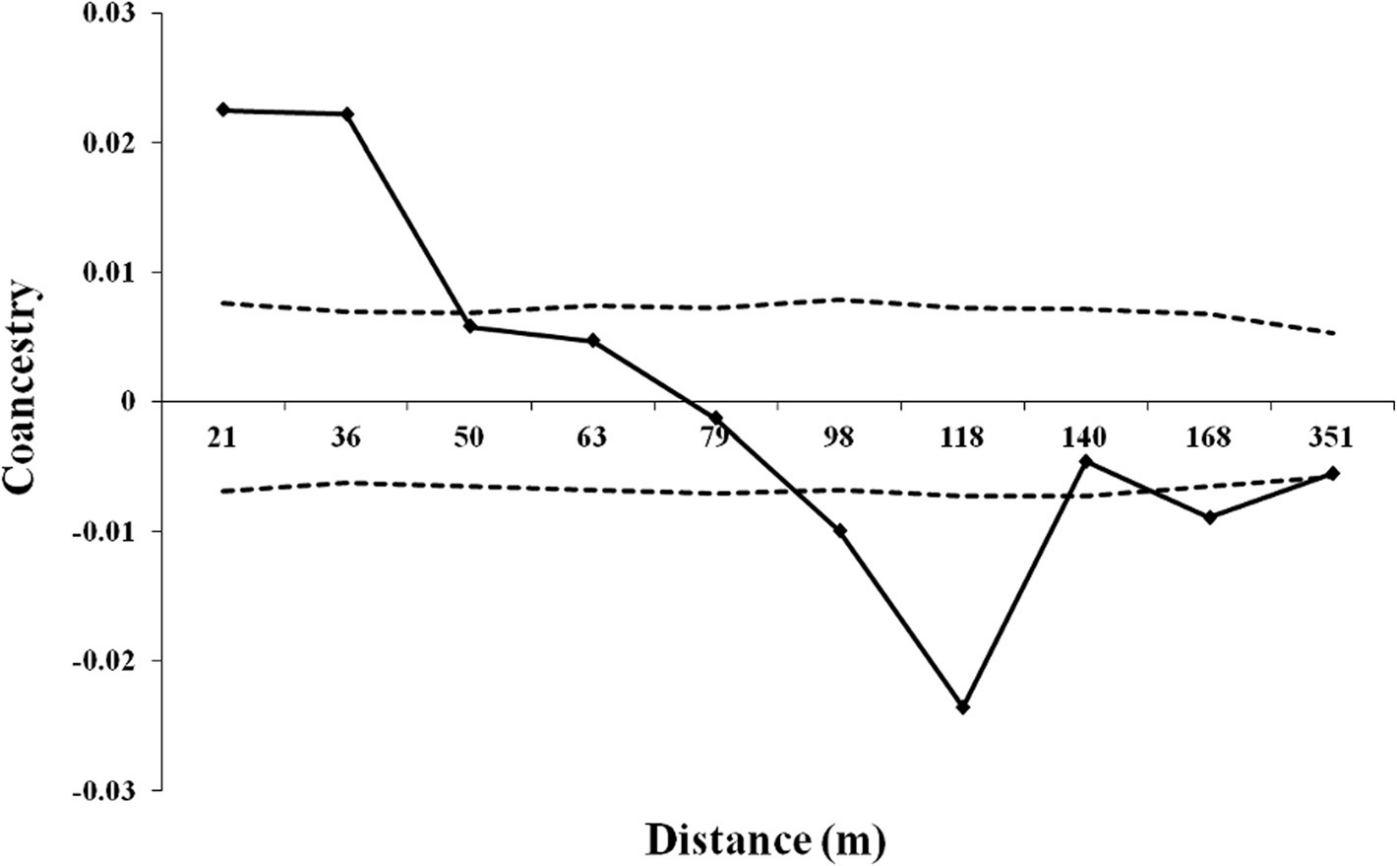
Correlogram of the coancestry coefficient (*θ*_*ij*_) of adult plants for ten distance classes. The continuous line represents the mean *θ*_*ij*_ value, the broken lines represent the 95 % confidence interval

The spatial genetic structure was significant up to 45 m (Fig. 1), suggesting that near neighbor adult plants are relatives. Above this distance the *θ*_*ij*_ values were not significantly different from zero or significantly lower than zero. The slope of the *b*_*k*_ regression of the pairwise coancestry coefficient over the logarithm of spatial distance scale (0 – 351 m) was significantly negative (*b*_*k*_ = -0.014), showing a seed dispersal pattern of isolation by distance. The *S*_*p*_ statistic was 0.0139.

### Effective population size

The group coancestry coefficient (Θ) for adults was 0.023, suggesting that the expected rate of inbreeding by mating among relatives is very low (<3 %). The estimated effective population size indicates that the 124 adult plants correspond to 22 (*N*_*e*_) unrelated and non-inbred individuals.

### Pollen flow, *dispersal patterns*, *and dispersal kernel estimation*

The combined probability of exclusion of the second parent (Table 1) was high (*P*_*2p*_ = 0.99608) in the paternity analyses. This indicates that pollen flow may have been overestimated because the higher number of pollen donors has been attributed for seeds using the delta statistic, and some true fathers may not have been assigned due to restrictions of this statistic. Among the sample of 120 offspring, 109 (90.8 %) were identified as being sired by an individual from within the sampled population. The other 11 offspring were probably sired by pollen donors from other populations, suggesting a 9.2 % of pollen immigration rate (Table 2). The 109 offspring were apparently generated by 56.4 % of the adult plants (70 of the 124 reproductive trees). No selfed offspring were detected, indicating a zero selfing rate.

**Table 2:** Pollen flow and dispersal in an *Astrocaryum aculeatum* population

Mother plants	M 04	M 05	M 06	M 07	M 15	M 37	M 40	M 41	M 45	M 47	M 48	M 49	Total
Np	15	2	7	14	10	8	8	15	13	2	14	12	120
Nm	1	-	3	-	2	1	1	-	-	-	1	2	11
m_p_ (%)	6.7	0	42.8	0	20	12.5	12.5	0	0	0	7.1	16.7	9.2

*Np* number of offspring genotyped per mother plants, *Nm* number of offspring with pollen donors located outside of the population, *m*_*p*_ percent of pollen flow

**Fig. 2:**
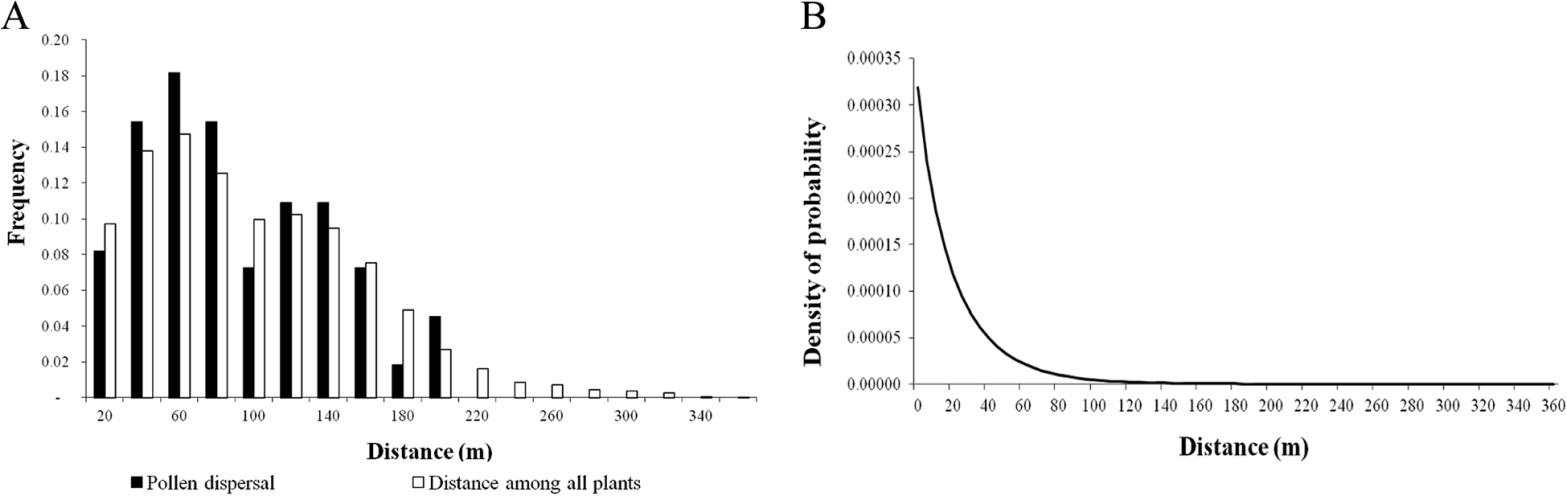
Pollen dispersal. **a** Effective frequency of pollen dispersal distance and the distance between the pollen donors and mothers plants in the studied *Astrocaryum aculeatum* population. **b** Estimated pollen dispersal kernel. Scale and shape parameters estimated using the neighbourhood model [54]

The pollen dispersal distance (δ) ranged from 3 to 194 m, with a mean of 81 m (standard deviation of 49 m) and a median of 70 m (Fig. 2a). In the study area, the estimate of the correlation coefficient among the number of offspring fertilized by pollen of male genitors (adult plants genotyped in this study) and the distance between mother plants was high and significantly different than zero (*R*^*2*^ = 0.74, *p* < 0.05). This suggests that the distance between plants had an impact on the mating probability. A Kolmogorov-Smirnov test was not significant (D = 0.064, *p* = 0.82; Fig. 2a), indicating that the spatial distance between trees explains the observed pattern of pollen dispersal. The mean effective pollination neighborhood area was 1.51 ha and the average effective pollination ratio of pollen dispersal was 69 m.

Pollen dispersal kernel for *A. aculeatum* was a slightly fat-tailed dispersal (Fig. 2b), indicating a high probability of long-distance dispersal of pollen (immigration). We also found absence of selfing (s = 0.0), pollen immigration rate of 0.098, mean distance of pollen dispersal of 90 m, dispersal kernel scale (a = 20.0739 m) and a slightly fat-tailed shape (b = 0.9).

## Discussion

### Genetic diversity

This is the first population genetic study in *A. aculeatum*, aimed at understanding gene flow in a natural population exploited through extractivism. The results show that this harvest practice has not had a negative effect on the genetic diversity of *A. aculeatum* offspring. By examining the mean number of alleles per locus (*A*), observed (*H*_*o*_) and expected (*H*_*e*_) heterozygosities, it was concluded that the levels of genetic diversity are similar between adults and offspring.

The great majority of the private alleles were found in the adults, suggesting that genetic drift may have occurred during the reproductive events, since not all of the alleles observed in the adults were transmitted to the offspring. Genetic drift is the random change in allele frequency that occurs because gametes transmitted from one generation to the next carry only a sample of the alleles present in the parental generation, and is more significant in small populations. In this species, female flower anthesis takes place in the evening and flowers remain viable for 24 h. Male flowers start their anthesis after female flowers and remain viable for only six hours [19]. *A. aculeatum* is a monoecious species with protogyny, without overlapping of sexual phases [19] and, consequently, expected to be predominantly outcrossing [20]. This was confirmed by the paternity analysis, which did not identify any instance of self-fertilization.

### Spatial genetic structure

The spatial genetic structure detected among the adults suggests a seed dispersal pattern of isolation by distance. Thus, near neighbor plants are probably relatives. Spatial genetic structure results from to the seed and pollen dispersal near the mother plant. The seeds from *A. aculeatum* are primarily dispersed by gravity, usually concentrated near the canopy projection of up to 3.5 m [4]. Secondary dispersal by rodents, such as *Dasyprocta azarae* and *Myoprocta* sp., places the seeds close to the mother plants [21]. This may explain the observed spatial genetic structure. The results also showed relatively low levels of coancestry in the two first distance classes (0 – 21 and 21 – 36 m), these being expected values between first degree cousins (*θ*_*ij*_ = 0.0625). Comparing the *S*_*p*_-statistic with other species, the observed value (0.0139) is similar to those identified in populations with a high density of individuals, such as *Dicorynia guianensis* (*S*_*p*_ = 0.026), *Vouacapoua americana* (*S*_*p*_ = 0.012) [22], *Sanicula odorata* (*S*_*p*_ = 0.0181), and *Silene acaulis* (*S*_*p*_ = 0.0144) [23], showing that density is one of the most important factors determining the spatial genetic structure.

### Effective population size

The adult coancestry group (Θ = 0.023) suggests that under random mating a low level of inbreeding is expected (< 3 %) [24], in agreement with the estimated inbreeding in both adults and offspring. Due to the low Θ, the effective population size (*N*_*e*_ = 22) was low [25], showing a high proportion of related individuals within the population.

The cause of the high proportion of relatedness within the population is likely due to short distance seed dispersal, as shown by the analysis of the spatial genetic structure. The pollen immigration rate (9.2 %), however, may in the future increase genetic diversity and effective size and so offset the negative effects of genetic drift.

### Pollen flow and dispersal patterns

Pollen flow from outside of the population was moderate (9.2 %). This gene flow was similar to the ones observed in other tropical tree species of wind and animal pollinated populations, which are isolated by a distance superior to 1 km. For instance, a pollen immigration rate of 10 % was observed in a population of *Araucaria angustifolia* distributed in an area of 5.4 ha and isolated from other populations by more than 1.7 km [13]. In a 4.8 ha forest fragment isolated from the nearest individual by a distance of 1.2 km, pollen migration in *Copaifera langsdorffii* was estimated as 5 to 8 % [14, 24]. In other studies with non isolated populations or localized at distances lower than 1 km, the immigration rates were higher: 49 % in *Symphonia globulifera* [26], 61.3 % in *Theobroma cacao* [27] and > 38 % in *Hymenaea courbaril* [28]. Pollen immigration increases the genetic diversity and effective population size, due to the introduction of new alleles [24]. Thus, the result suggests that pollen immigration contributed moderately to the preservation of the genetic diversity in the *A. aculeatum* population.

Pollen dispersal reached long distances within the population (194 m), considering that the maximum distance between two plants was 211 m. However, pollen dispersal followed an isolation by distance pattern (Fig. 2a), shown by the high correlation (*R*^*2*^ = 0.74) between the number of seeds fertilized by pollen donors located close to the mother plants. There are no previous studies on the pollinators of *A. aculeatum*. However, floral morphology and phenology are similar to the congener *Astrocaryum vulgare* [29], suggesting that *A. aculeatum* has similar pollinators. *A. vulgare* is pollinated by bees of the species *Trigona sp*. and *Apis mellifera* (Hymenoptera) and Coleoptera belonging to the families Nitidulidae (*Mystrops* sp.) and Curculionidae (*Terires minusculus*) [29]. We observed the species *Trigona* sp. and *A. mellifera* visiting flower buds in pre-anthesis and anthesis of male flowers (floral damaging these parts in search of pollen). In addition, Coleoptera (*Mystrops* sp. and *Terires minusculus*) insects were observed both in male and female flowers of *A. aculeatum*. These insects have potential for long-distance pollen dispersal [30]. This could explain the observed high effective pollination area (1.51 ha).

The mean pollen dispersal distance of *A. aculeatum* (81 m) by insects pollinators was similar to that detected in high-density populations (> 5 tree/ha) [15]. In general, this distance is lower than 100 m. Pollen dispersal distance for high-density populations rarely exceeds 300 m in forests [15]. For example, in *Astrocaryum mexicanum* the average varies between 13 to 23 m [31], while in *Oenocarpus bataua*, a species that occurs in low-density populations, the estimated distances ranges from 113 to 1263 m [32]. In tree species from other families, pollination dispersal distances range from 28 m in *Theobroma cacao* [27], 65 m in *Himatathus drasticus* [33] and 66 to 94 m in *Copaifera langsdorffii* [14, 24, 34]. Our results showed that a higher frequency of short pollen dispersal distance relative to long pollen dispersal distance suggests that *A. aculeatum* is primarily an animal-pollinated species [15].

The exponential power distribution of inter-mate distances within our study plot had a similar behavior of a slightly leptokurtic shape (b = 0.9), indicating some long-distance mating events. Similarly, the dispersal kernel inferred for *A. aculeatum* using the spatially-explicit Neighborhood model showed a distribution with high probability of dispersal at low-distances. However, a very rapid decline in dispersal probability with increasing distance was observed. Similar long-distance dispersal events at pollination and overall pollen movement have been observed in recent studies (for example, with *Araucaria angustifolia* [13] and *Phoenix canariensis* [35]), suggesting a pattern in pollination events that may not be uncommon, especially amongst tropical species, because it showed a non-leptokurtic pattern [32].

The results observed in *A. aculeatum* would indicate that the genetic data used for the underlying model of dispersal, which typically assumes a long tail of dispersal [32] are in agreement to the model. The shape of the tail of the dispersal kernel (that is, whether thin- or fat-tailed) impacts the ultimate distribution of genetic diversity within and between populations. Most studies report that pollen dispersal kernels are fat-tailed in tree species [35–38]. This shows that the slightly fat-tailed dispersal distribution found in the sampled *A. aculeatum* is related to a few long distance dispersal events, due to the relatively low pollen immigration detected (9.8 %).

### Implications for conservation, cultivation of trees and seed harvesting

Our results have important implications for seed harvesting strategies for *ex situ* conservation and commercial reforestation of *A. aculeatum*. The presence of spatial genetic structure within the population indicates that seeds should be harvested from mother plants located at distances larger than 45 m from each other. This practice will limit the probability of collecting seeds from related plants, which would reduce the effective size of collected progeny. The inclusion of offspring from different and non-related mother plants in germplasm banks increases the effective size of the retained population [13]. However, considering that spatial genetic structure occurs in the studied population and 50 % of the pollen is dispersed below 70 m (median pollen dispersal distance), we can expect some levels of inbreeding in the sampled progeny arrays, originated from mating among relatives [13]. Some inbreeding may be eliminated in nurseries, by excluding seedlings with low growth and poorly developed. Therefore, the estimation of genetic parameters in progeny tests with seeds from this population must be adjusted to accommodate inbreeding.

## Conclusions

The studied population presents high levels of genetic diversity in both adults and open-pollinated seed, due to pollen flow. However, the effective size of the adult population is low due to the presence of spatial genetic structure caused by short seed dispersal distances. Also, pollen dispersal follows a model of isolation by distance. Consequently, most of the mating occurred among neighboring plants and open-pollinated seeds probably present some levels of inbreeding. It will be critical to repeat this study in other populations and/or the next reproductive events of the mother plants to ascertain the observed results.

## Methods

### Study area

**Fig. 3:**
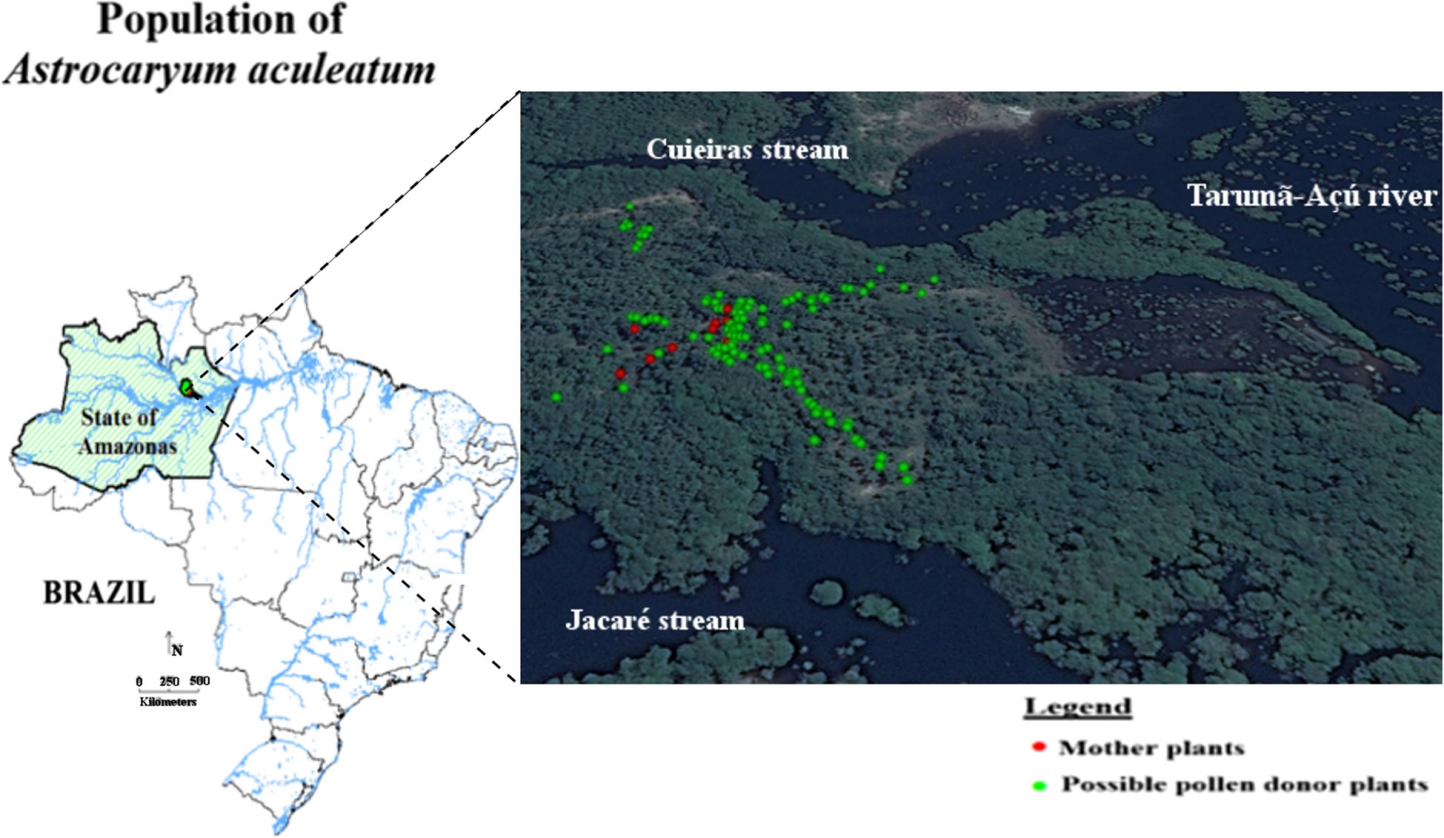
Collecting site from a harvested population of *Astrocaryum aculeatum* at the Najatuba property, in Manaus, state of Amazonas, Brazil. Map made with DIVA-GIS version 7.5 [55], showing a panoramic Google Earth Digital Globe 2014 of the study area

This study was undertaken in a natural population of *A. aculeatum*, located in the lot number 12 of the Manaus Agrarian Project, in a rural property named “Natajuba” (latitude -02°53’27.9”S and longitude -60°06’08.2”W) in the state of Amazonas, Brazil (Fig. 3). The region is characterized by a tropical forest climate (Af type), according to the Koppen-Geiger world map of climate classification [39]. The population is bordered to the East by the hydrographic basin of the Tarumã-Açu river, and to the West by a native forest that is part of the legal reserve of the “Natajuba” property and other properties within the Manaus Agrarian Project. North and South boarders are delimited by the rivers “Cuieiras” and “Jacaré”, respectively. Each side of the streams has a riparian forest of width larger than 200 m. Two other natural populations of *A. aculeatum* are located 400 m from the northern and more than 1,000 m from the southern boarders of the population studied here. Additional *A. aculeatum* populations are located at distances higher than 3,000 m. The population used in this study has been exploited continuously since 1996 to provide *A. aculeatum* fruits to the Manaus market. This population is also part of the *in situ* conservation of the superior germplams program from Embrapa Western Amazonia, within the project “Research, development, and innovation in oil producing palm plants and the economical use of by-products and residues”, PROPALMA (Embrapa-Propalma).

### Sampling

In March 2011, 12 mother plants of *A. aculeatum* with maturing fruits generated by open-pollination were identified in the population area [4]. Twenty-five fruits were collected from each mother plant and placed within properly identified polyethylene bags and taken to the seed laboratory of the Western Amazonia Embrapa, in Manaus (Brazil). The pulp of each fruit was removed and the seeds obtained were dried at a temperature of 30 °C up to the point where the moisture content was 14.5 %, allowing the separation of the seed tegument by mechanical breakage [3]. Soaking and germination processes were immediately carried out [3]. The offspring germination phase was conducted during three months in the greenhouse. A total of 120 offspring from 12 mother plants of *A. aculeatum* was obtained. The number of offspring obtained per plant varied from 2 to 15. The distance between the mother plants in the area ranged from 6.2 to 83.2 m, with a mean of 37.7 m and a median of 37.1 m. Based on the geographic distribution of the mother plants, the center point between them was identified. From this central point four transects were established to conduct sampling of adult plants that are potential parents (pollen donors) of the offspring obtained from the mother plants. The end of each transect was established when there were no more plants within a distance of 100 m. Each transect was 10 m wide. These transects were extended to the Northeast, Southeast, Northwest and Southwest directions. In the four transects, 112 possible pollen donor plants were sampled. The distance between possible pollen donors varied from 0.5 to 351.4 m, with a mean of 89.6 m and a median of 78.9 m. The geographic position of each adult plant (pollen donor and mother plant) was determined by using a global positioning system (GPSmap 60CSx - GARMIN).

Leaf samples from 120 offspring, 12 mother plants and 112 potentials parent plants (pollen donors), were collected and stored in silica gel at -20 °C in the Laboratory of Molecular Biology at the National Institute of Amazonian Research (LTBM-INPA). Total DNA was extracted according to the cationic detergent protocol CTABLE 2× (Cationic Hexadecyl Trimethyl Ammonium Bromide) [40] and quantification was performed according to Ramos et al. [20].

This research was supported by the norms of Resolution 21 - 31 August 2006 - of CGEN (*Conselho de Gestão Do Patrimônio Genético* - *Ministério do Meio Ambiente*) (http://www.mma.gov.br/estruturas/sbf_dpg/_arquivos/res21cons.pdf). Material collection was registered in SISBIO (*Sistema de Autorização e Informação em Biodiversidade*/*Instituto Chico Mendes de Conservação da Biodiversidade* - *ICMBio*/*Ministério do Meio Ambiente* - *MMA*), voucher number 39950-4. A sample of the taxon *Astrocaryum aculeatum* G. Meyer (tucumã-do-Amazonas) was deposited in the INPA herbarium under No. 246369.

### Microsatellites amplification

In this study, 12 microsatellite loci developed for *A. aculeatum* (Aac01, Aac02, Aac03, Aac04, Aac06, Aac07, Aac09, Aac10, Aac11, Aac12, Aac13 and Aac14) were used [41]. These microsatellites were amplified by polymerase chain reaction (PCR) using the Veriti Thermal Cycler (Applied Biosystems) in a total reaction volume of 10 μL, containing 10 ng genomic DNA, 1× buffer (10× standard *Taq* reaction buffer), 210 μM of each dNTP, 1.5 mM MgCl_2_, 0.16 μM of forward and M13 labeled primers (FAM or NED dyes) [42], 0.32 μM of reverse primers, 1.05 U *Taq* DNA polymerase (Invitrogen), and 3.49 μL of ultra pure water. The amplifications *via* PCR occurred in two phases, the first being specific for the primers and the second to connect the M13. The first stage began by stabilizing the temperature at 68 °C for 2 min and at 92 °C for 30 s, followed by 30 cycles (30 s at 92 °C for denaturation process, 35 s at the primer-specific annealing temperature {Table 1 of [41]}, and 30 s at 68 °C {72 °C for Aac07 and Aac11} for extension); the second step consisted of 15 cycles (30 s at 92 °C, 30 s at 53 °C, 30 s at 72 °C) and a final extension at 72 °C for 15 min followed by a period of 15 min at 68 °C [20, 41].

Amplification products were checked by electrophoresis on 1.5 % agarose gels stained with GelRed (Biotium) in 1× TBE buffer (pH 8.0). Amplified products of the PCR were submitted to an automatic DNA analyzer by capillary electrophoresis in the ABI 3130XL Genetic Analyzer (Applied Biosystems). The ET-550 ROX size standard (GE Healthcare) was used to determine the size of the alleles. Amplified fragments were observed and analyzed with the GENEMAPPER v4.0 software (Applied Biosystems).

### Statistical analysis

#### Analysis of genetic diversity and fixation index

Genetic diversity was determined to compare adults and offspring, using the indexes total number of alleles over loci (*k*), average number of alleles per locus (*A*), number of private alleles in each generation (*A*_*p*_), and the observed (*H*_*o*_) and expected (*H*_*e*_) heterozygosities. These indexes were estimated using the GDA program [43]. Inbreeding was estimated using the fixation index (*F*). To test whether the *F* values were statistically different from zero, 1,000 Monte Carlo permutations of alleles among individuals, associated to a *Bonferroni* correction (95 %, α = 0.05), were obtained using SPAGeDi 1.3 [44]. To investigate if the mean values of *A*, *H*_*o*_, *H*_*e*_ and *F* were significantly different between adults and offspring, the Student *t*-*test* was used, with a prior verification of the homogeneity of variances of the two groups, using a Fisher’s *F*-*test*. These analyzes were performed using the *var.test* and *t.test* functions of R package from the R project [45].

#### Analysis of the spatial genetic structure

The intrapopulation spatial genetic structure was studied using the mean coancestry coefficient (*θ*_*ij*_) between pairs of adult plants, calculated according to Loiselle et al. [46] and using the SPAGeDI program. To visualize the spatial genetic structure, values of *θ*_*xy*_ were plotted against ten distance classes with the same number of pairwise individuals. In order to verify whether the spatial genetic structure had a significant deviation from a random structure, the CI of 95 % was calculated for each *θ*_*ij*_ observed value and each distance class, using 10,000 Monte Carlo permutations of individuals among different distance classes. To compare the spatial genetic structure with other studies we estimated the *S*_*p*_ statistic: *Sp* = − *b*_*k*_/(1 − *θ*_1_) [23], where *θ*_*1*_ is the average coancestry coefficient calculated in the first distance class (0 to 21 m), and *b*_*k*_ is the slope of the regression curve in relation to the logarithm of the spatial distance (up to 361 m). To test the intensity of SGS, the spatial position of the individual was permutated 10,000 times to obtain the distribution frequency of *b*_*k*_ where the null hypothesis states that *θ*_*1*_ and ln *d*_*xy*_ are not correlated (*d*_*xy*_ is the spatial distance between individuals *x* and *y*). These analyses were run using SPAGeDI 1.3 program.

#### Analysis of the group coancestry and population effective size

The group coancestry (*Θ*) [47] was estimated for the adult plants from pairwise coancestry coefficient between all pairs of individuals (*θ*_*ij*_), using the estimator described in Loiselle et al. [46], implemented in the SPAGeDI program: *Θ* = [0.5*n*(1 + *F*_*p*_) + ∑_*i* = 1_^*n*^∑_*j* ≠ *i*_^*n*^*θ*_*ij*_]/*n*^2^, where *n* is the number of sampled individuals, *F*_*p*_ is the inbreeding coefficient of the population, estimated from the fixation index (negative value are assumed as zero). The effective population size (*N*_*e*_ was calculated following Cockerham [48] from the variance of gene frequencies due to genetic drift (*σ*_*p*_^2^ = [(*n* − 1)/*n*)*Θ* + (1 + *F*)/2*n*]*p*(1 − *p*), where *n* is the sample size, *p* is frequency for a given neutral allele and *F* is the average inbreeding coefficient. In an idealized population under random mating, *σ*_*p*_^2^ value for a group of *n* individuals is *σ*_*p*_^2^ = *p*(1 − *p*)/2*n* and as in a idealized population there is not related and inbred individuals, the term *n* can be substituted by *N*_*e*_ : *σ*_*p*_^2^ = *p*(1 − *p*)/2*N*_*e*_. Thus, we can equate both *σ*_*p*_^2^ expression and derive the variance effective population size, ${N}_e=\frac{0.5}{\varTheta \left(\frac{n-1}{n}\right)+\frac{1+F}{2n}}$.

#### Analysis of the pollen flow, dispersal patterns and dispersal kernel estimation

For the paternity analysis, the CERVUS 3.0.3 program [49] was used, based on a categorical maximum likelihood method. The offspring paternity was determined by the Δ estimated statistic, calculated using simulations, considering 10,000 repetitions (simulated for the offspring), zero error rate at the loci (0.00) and all the 124 reproductive palm trees (112 adults + 12 mother plants) as pollen candidates for the offspring (60 % of sampled pollen donors collected in the study area). We adopted the confidence levels of 80 % as suggested by Marshall et al. [49] for the paternity assigned. Self-fertilization was also considered as a possibility and was estimated. The pollen immigration rate (*m*_*p*_) within the area was estimated as the number of offspring for which no father was assigned in the sampled area, divided by the total number of sampled offspring. The pollen dispersal distance for each progeny was calculated as the distance between the seed trees and the putative pollen donors by the Euclidian distance between two points. To verify whether the reproduction patterns were due to the distance between plants, the frequency of pollen dispersal curve was compared with the spatial distance among all plants using the Kolmogorov-Smirnov test [50]. The effective pollination neighboring area (*A*_*ep*_) was calculated assuming a circular area around a central seed tree, by *A*_*ep*_ = 2*πσ*_*p*_^2^ [51], where *σ*_*p*_^2^ is the axial variance of the pollen dispersal.

The combined probability of exclusion of second parent, *P*_*2p*_ [52], was estimated with the NM+ program [53]. We also estimated pollen flow, selfing and pollen dispersal distance assuming an exponential power dispersal kernel [37], implemented in the NM+ program [53]. This program is based on neighborhood model [54]. In this model, the pollen dispersal distance and patterns are not derived from individual paternity assignments, as in the case of Cervus, but indirectly from a spatial explicit mating model. The model considers that paternity of an offspring may result from: i) self-fertilization with probability *s*; ii) migrant pollen from outside the plot, with probability *m*_*p*_, or iii) outcrossing with a male located within the plot, with probability 1-*s*-*m*_*p*_ [54]. The NM+ was matched with initial settings using categorical paternity assignment for our study plot. The neighborhood parameter was set to ‘infinite’ to include all sampled adults in our study plot as the neighborhood size [53]. Pollen dispersal was modeled using the exponential-power family parameter [37, 53] with estimates given of the scale (*a*) and shape (*b*) parameters from which the average distance of pollen dispersal (δ) is estimated.

### Ethics

Not applicable.

### Consent to publish

Not applicable.

### Availability of supporting data

The data sets supporting the results of this article are included within the article.

## Abbreviations

*σ*_*p*_^2^: Variance of gene frequencies due to genetic drift
*A*: Average number of alleles per locus
a: Dispersal kernel scale
*A*_*ep*_: Effective pollination neighboring area
*A*_*p*_: Number of private alleles
b: Shape parameters
*b*_*k*_: Slope of the regression curve in relation to the logarithm of the spatial distance
*C*_*gf*_: Cryptic gene flow
D: Kolmogorov-Smirnov test
*d*_*xy*_: Spatial distance between individuals *x* and *y*
*F*: Fixation indexes
*H*_*e*_: Expected heterozygosity
*H*_*o*_: Observed heterozygosity
*m*_*p*_: Pollen immigration rate
*n*: Sample size
*N*_*e*_: Effective population size
*n*_*p*_: Number of candidate parents
*p*: Frequency for a given neutral allele
*P*_*2p*_: The combined probability of exclusion of the second parent
*R*^*2*^: Correlation coefficient among the number of offspring fertilized by pollen of male genitor and the distance between mother plants
s: Selfing rate
SGS: Spatial genetic structure
*S*_*p*_: Statistic which measure the extension of spatial genetic structure in the fist distance class
δ: Pollen dispersal distance
Θ: The group coancestry coefficient
*θ*_*1*_: Average coancestry coefficient calculated in the first distance class
*θij*: Mean coancestry coefficient
